# Obstetrics

**Published:** 1910-12

**Authors:** Walter C. Swayne


### OBSTETRICS.

**Uterine Fibroids.**—The life histories of patients who suffer from large uterine fibroids are in many cases interesting studies. Few practitioners of over twenty years' standing have not had experience of one at least of these, and many have been able to observe several.

Any attempt to present complete records of a number of cases of fibroids, especially when the histories are fairly complete, must be of considerable value, and an attempt to collect a long series of such cases by obtaining information from several observers would give some extremely interesting information as to the results to the patients of non-interference.

A. J. Wallace[1] publishes the after-history of ten of such cases, giving in each case diagrams representing the size and position of the tumour in various stages of development or retrogression. The tumour in every case was large, and produced symptoms such as pain and hemorrhage.

In eight out of the ten cases observed incomplete shrinking took place, and in three cases the decrease in size was very rapid. It would seem reasonable to suppose that in these

1 *J. Obst. and Gynæcol. Brit. Empire*, 1910, xviii. 20.

cases the rapid diminution was the result of the disappearance of œdema. In one case the disappearance took place before the menopause; in three shrinking commenced before the menopause; in three at the menopause, and in three after its onset. It is doubtful whether in any one of the ten cases the presence of the tumour had any effect on the condition of the heart, although the hemorrhage due to the tumour apparently affected the heart unfavourably.

The history after the menopause showed good health in four out of the ten. In one the menopause has not yet occurred, but her health is good. Of the remaining five, one died after years of invalidism, three remain invalids, and one, although otherwise fairly well, is blind.

These histories do not predispose to a favourable view of the results of palliative treatment. In only one case among those six in which a state of moderate comfort was reached, could it be said that the patient gained from non-interference moderate comfort was only arrived at after years of suffering and broken health, while even this cannot be permanently depended on when the degenerative changes possible, even after the menopause, are borne in mind.

**Fibromyoma in Pregnancy.**—At the meeting of the British Medical Association on July 26th to 31st a discussion took place on this subject, which was initiated by Dr. Walter Tait. Many speakers related their experiences as regards this complication. Among the accidents described were the following:—Premature interruption of pregnancy, necrobiotic changes of the tumour, difficulties in the removal of the placenta, ruptured uterus, unbearable pain, axial rotation of the uterus, rapid increase in the tumour with abdominal pressure symptoms, delivery obstructed by the presence of a tumour in the pelvic canal. The opinion of the majority of the speakers showed that, although in a large proportion of cases fibroids occurring as a complication of pregnancy do not cause serious or dangerous symptoms, these latter do arise in a certain number of cases, and may require to be dealt with by operative measures. Several cases were reported where the symptoms present necessitated the removal of the uterus by sub-total hysterectomy, and many others in which the tumour was removed by myomectomy without interfering with the course of pregnancy, delivery taking place at term. The general trend of opinion as regards operative interference seemed to be that laparotomy having been decided on, the operator should in all cases be prepared to remove the uterus and tumour together, but where possible, operative interference should be restricted to the removal of the tumour itself by enucleation, leaving the uterus intact. Even tumours with broad attachments can be removed by this method without terminating pregnancy and one case has been described in which the uterine mucosa was exposed by the removal of the tumour, but the pregnancy still continued. The chief danger is the hemorrhage from the raw surface left by the removal. This can generally be dealt with by suturing in layers. In any case of unusual difficulty or danger the removal of the uterus can always be adopted as a possible termination.

**Myomata in Pregnancy, Labour and Childbed, and their Influence on Conception.**—F. von Winckel's paper[1] deals chiefly with a monograph by Theodor Landau on myomata in pregnancy, labour and childbed, and in the first place he shows the rapid diminution in the mortality rate of operations for their removal; thus in 1885 the mortality was between 30 and 40 per cent., twelve years later 5.6 per cent., seven years later 4.6 per cent. In the last five years 283 operations by the brothers Landau resulted in two deaths, less than 1 per cent. This is in accordance with the experience of others. The constantly diminishing mortality from the severe operations necessitated for the relief of these conditions demonstrates the decrease in the danger due to improved technique.

Von Winckel states that Landau, contrary to the opinions of other observers, finds that uterine myoma is a disease of youthful sexual life. Ten per cent. of the cases operated on by them were found in women between twenty and thirty years old. Again, with regard to the fertility of women suffering from myoma, Landau found that the majority of cases (about 72 per cent.) had been fertile, and of the remainder only a few were suffering from conditions which would have made pregnancy unlikely. It seems evident, therefore, that although relative sterility has been found by a large number of observers to accompany the presence of large fibroids, this by no means necessarily follows, and pregnancy is always possible, provided that the continuity of the uterine cavity is not definitely interfered with. Consequently it is found, as is is to be expected, that quite a fair proportion of pregnancies occur in myomatous uteri, and that in a certain proportion of these cases symptoms due to the presence of myoma in pregnancy are likely to be met with.

Krusen[2] enumerates the dangers which may arise when fibroid tumours complicate pregnancy and the puerperium. The growth may rapidly increase in size; this is due rather to œdema than to pure hypertrophy. Twisting of the pedicle may occur in pedunculated growths, leading to gangrene and peritonitis. Malpresentations occur in about 49 per cent. of

[1] *München. Med. Wchnschr.*, 1910, lvii, 1698. Quoted in *J. Obst. and Gynæcol. Brit. Empire*, 1910, xviii. 282.

[2] *Am. J. Obst.*, 1910, lxi. 458. Quoted in *J. Obst. and Gynæcol. Brit. Empire*, 1910, xvii. July.

cases. The presence of adhesions may produce severe symptoms, similar to those found in impaction of the retroverted gravid uterus. Tubal pregnancy may be caused by pressure of the fibroid on the tubes. Normal involution is often seriously impeded, hemorrhage and septicæmia both being liable to occur. This is especially the case after abortions. Rupture of the uterus due to thinning of the wall in the neighbourhood of the tumour is possible. Aggravated pressure symptoms and degeneration of the cardiac muscle, and also of the renal and hepatic epithelium, are also met with. Placental adhesions and ineffective uterine contractions are shown to be more frequent. The presence of the tumour may give rise to displacements of the uterus, and occasionally lead to axial rotation.

Krusen does not favour myomectomy during pregnancy, and advises supra-vaginal hysterectomy where urgent symptoms due to pressure are observed, or where the growth is apparently the subject of degenerative changes.

E. H. Robinson[1] deals with the question of myomata which become separated from the uterus and form attachments to other organs, from which they derive their blood supply. He shows that these separations take place first by uterine contractions; secondly, by growth along the line of least resistance, and thirdly on account of unequal blood supply to different parts of the tumour. These considerations would lead, as regards the first, to expulsion in a certain number of cases of submucous fibroids; but the same conditions occurring in the sub-serous variety will lead to the formation of the so-called parasitic myomata. This condition is a comparatively rare one, as Kelly and Cullen only met with two instances in 1,764 cases.

The process of separation is necessarily associated with marked circulatory disturbance in the tumour, its attachment to the uterus becoming more and more circumscribed, with a proportionately diminishing blood supply from the uterus, until this finally ceases altogether. Usually degenerative changes occur before separation is complete, and the tumour then becomes a foreign body lying within the peritoneal cavity. Adhesions then form to the neighbouring organs, such as the omentum and intestines, and a new vascular supply is obtained from these, consisting of thin-walled, much enlarged omental vessels spread out over that portion of the tumour which is adherent to the omentum, with much resulting risk to the patient and the possibility of hemorrhage, which is apt to be excessive and stopped with great difficulty, occurring during an operation for its removal.

Richardson relates a case of the kind. A multipara, age 38, was admitted complaining of pain and swelling in the lower

[1] *Surg. Gynec. & Obst.*, 1910, xi. 297.

abdomen. A firm tumour with an irregular surface was felt situated in the region of the umbilicus about the size and shape of the normal kidney. The uterus was found to be about three times the normal size, its enlargement being due to a fibroid in the fundus. No connection could be detected between the movable tumour above and the uterine fibroid. The abdomen was incised, and the movable tumour at once presented. It was extremely dark in colour, with a lobulated surface, and lay half buried in loops of small intestine, the anterior surface adherent at one point to the parietal peritoneum. From its colour it was obvious that degenerative changes were taking place. The tumour was removed, leaving a raw surface over the small intestine, which was covered in with peritoneum after the various adhesions of the small intestine had been separated. Convalescence was uneventful. When cut into the parasitic tumour was found to be a degenerated myoma which had obviously separated from its attachment to the uterus.

Stone[1] reports on 150 cases of fibroids operated on by him, and in addition gives numerous extracts taken from the records of similar cases published by operators. Among some of the interesting facts recorded by him are some relating to the association of malignant disease and fibromyoma. He is of opinion that five per cent. of all tumours diagnosed as fibromyoma before operation would prove to be malignant.

Fehling, Hofmeier, Winter, Scharlieb, Freund and Haultain have reported between them 3,661 cases of fibromyoma in eighty-one, or 2.2 per cent., of which carcinoma was found. This figure would appear to be the average.

As regards sarcoma, Winter found sixteen in 500 cases of fibromyoma, or 3.2 per cent. Stone himself met with three cases in his series of 160. Bland Sutton, it should be noted, however, denies that sarcomatous degeneration occurs in fibromyoma, and states that these are sarcomatous from the beginning. (The writer has met with three cases of sarcoma in, and one of adenocarcinoma with, fibroids in the last three years.)

The mortality rate of fibromyomata when not operated on is so variously stated by different observers that it is difficult to reconcile such figures as those of Noble (thirty to thirty-eight per cent.), Keith (ten per cent.), and McDonald (eight per cent.) with those of Roger Williams (.05 per cent., or one in 2,000). The mortality of hysterectomy for fibroids is shown to have fallen enormously during the last few years. Stone, however, remarks very justly that a low mortality rate may mean a careful selection of cases and the exclusion of those in obviously bad health.

It would appear that the mortality rate after operations for

[1] *Am. J. Obst.*, 1910, lxii. Sept.

fibromyoma might fairly be expected to vary from about three per cent. for selected cases to nine or ten per cent. if a large proportion of complicated cases are included.

Savill[1] relates some experience of the treatment of a case of fibromyoma with X-rays. She found that although the size of the tumour did not diminish, her patient's health, much deteriorated by hemorrhage, improved owing to the cessation of the latter after over forty applications of the rays in small doses. She quotes Bordier's experience with regard to the reduction of these growths after a prolonged course of X-ray treatment, and concludes that in her opinion this method of treatment, judging from her case, and those reported by other workers, has a considerable future.

The following conclusions may be drawn from the above :—

(1) That non-operative treatment in the hope of the disappearance of the tumour after the menopause, means years of invalidism, without the certainty of restoration of the patient's health, and liability to degenerative and possibly malignant changes.

(2) The possible development of malignant disease is a very real danger in this case, although the percentage of such cases is not large.

(3) Fibroids in themselves cause a definite rate of mortality.

(4) Fibroids occurring as a complication of pregnancy may cause severe and even very dangerous symptoms, and that when these occur operative treatment is indicated.

(5) When, under these last circumstances, interference is indicated, myomectomy should be performed if possible, but in a large number of cases total or sub-total hysterectomy will be required.

Walter C. Swayne.

